# The Study of Adolescent Resilience (SOAR): a research protocol

**DOI:** 10.3389/frcha.2024.1346726

**Published:** 2024-03-19

**Authors:** Karen Tannenbaum, Hope Seib McMaster

**Affiliations:** ^1^ Military Population Health Directorate, Naval Health Research Center, San Diego, CA, United States; ^2^ Performance and Readiness Division, Leidos, Inc., San Diego, CA, United States

**Keywords:** military, youth, adolescents, psychosocial adjustment, cohort, health

## Abstract

**Background:**

Adolescence is a particularly sensitive period of development for military-connected youth, given the socioemotional and physical changes that occur against the backdrop of the military career of their parent(s). Military-connected adolescents face unique stressors relative to their civilian counterparts, such as military relocations, parental absence due to deployments and trainings, and parental military-related physical and mental injury. These stressors may change family dynamics and disrupt social support networks, which can have lasting implications for adolescent health and well-being. At present, very little is known about how the current generation of military-connected adolescents are faring regarding their psychological and physical health, academic achievement, and educational or career aspirations. As part of the Biden-Harris Administration's commitment to military families, the U.S. Department of Defense pledged to address these major knowledge gaps by supporting the Study of Adolescent Resilience (SOAR). SOAR's longitudinal design provides a powerful tool for evaluating the effects of military family life on adolescent well-being into early adulthood.

**Methods and design:**

SOAR is embedded within the larger Millennium Cohort Study, the first U.S. population-based prospective study to investigate long-term health effects of military service among active duty service members. Nearly 40,000 Millennium Cohort participants with adolescent children were invited to complete a web-based self-report survey that included items across interpersonal, socioenvironmental, and health domains, among others. Military parents provided referral information for their adolescent child and another primary parental figure, when available, to be invited to SOAR. This approach allowed survey data to be linked between these three family members (service member, spouse, and adolescent) to promote a comprehensive, family systems-based understanding of military-connected adolescent experiences and determinants of health, risk, and resilience.

**Discussion:**

Research findings will shed light on the enduring impact of military life on adolescents and the nature of associations between military-specific experiences and psychosocial health and well-being. Further, this research will assess modifiable risk and protective factors that may elucidate differences in military-connected adolescent psychosocial development and physical health, academic achievement, and educational and military career aspirations over time.

**Conclusions:**

Findings gleaned from this research will be used to inform existing policy and programs designed to promote adolescent resilience.

## 1 Introduction

There are over 1.6 million children with a parent in the U.S. military, of whom about one quarter are adolescents between the ages of 12 and 18 years (1). Adolescence is a sensitive period of development in many areas that are critical to healthy adult functioning, such as the formation of a strong identity and independent sense of self (2, 3). Adolescents’ relationships with their parents transform during adolescence, and peers become more influential as adolescents spend more time away from home (4, 5). These social changes intersect with intense biological changes, including hormonal shifts that contribute to the development of mental health problems such as depression and anxiety, with rates peaking as adolescents approach young adulthood (6). Specifically, the National Institute of Mental Health reported rates of mental health conditions among adolescents in the U.S. general population of 31.9% for general anxiety disorders (38.0% for females, 26.1% for males), 17.0% for major depressive episode (25.2% for females, 9.2% for males), 8.7% for attention-deficit/hyperactivity disorder (13.0% for males, 4.2% for females), and 5.0% for posttraumatic stress disorder (PTSD; 8.0% for females, 2.3% for males) (7–10). In addition, suicide is the second leading cause of death for adolescents, behind unintentional injury, with the age-adjusted suicide rate increasing 35.2% since 2000 from 10.4% to 13.5% suicides per 100,000, which is nearly four times higher for males (22.0 per 100,000) than females (5.5 per 100,000) (11). Data from the national 2021 Youth Risk Behavior Survey, the first since the beginning of the COVID-19 pandemic, reveal that poor mental health and suicidal thoughts and behaviors are increasing for nearly all groups of youth and that female students are faring more poorly than male students (12). Given these alarming trends before and over the course of the COVID-19 pandemic, the American Academy of Pediatrics, the American Academy of Child and Adolescent Psychiatry, and the Children's Hospital Association recently joined together to declare a national state of emergency in children's mental health (13).

For military-connected adolescents, these profound physical, social, and emotional changes occur against the backdrop of the military career of their parent(s). Adolescents who grow up in military families face unique stressors compared with civilian youth, such as unexpected and frequent military relocations, changes in schools and peer groups, and disruptions in family dynamics due to parental absence (14–16). For example, Chandra and colleagues (17) found a linear association between the greater number of months a parent was deployed and adolescents' number of difficulties. Research with small, nonrepresentative samples of mostly Army-connected youth has examined youth perceptions of parental deployment and found that adolescents reported changes in their academic performance as well as an awareness of the dangers associated with deployment (18). This cognitive awareness may lead to ingrained thought patterns in youth that contribute to increased risk of anxiety and depression (19–21).

Additionally, military stressors may affect adolescents indirectly through their impact on their parents' marital relationship, the quality of the parent–adolescent relationship, and parenting behaviors. Namely, the military parent's exposure to high operational tempo and combat increases the risk for negative physical and psychological health outcomes (e.g., PTSD, depression, substance use, traumatic brain injury, sleep disturbance, physical injuries) related to deployment (22–24). Parental PTSD has been linked to increased risk for family violence, poor parenting behaviors, and disengagement with children (17, 19, 25, 26), with PTSD symptoms often increasing during the postdeployment period (27). Relatedly, the postdeployment adjustment of children is characterized by increased risk for internalizing and externalizing problems (19, 28).

Nevertheless, some military-connected adolescents seem to exhibit resilience and, in fact, may flourish relative to stress experiences. In a small study of adolescents observed during a parental deployment (18), researchers found that most adolescents took on additional household responsibilities while their parent was deployed, and older adolescents also appeared to have a better understanding of the sacrifices required for their parent's military career; this ability to process stress compared with younger adolescents may mitigate detrimental aspects of stress (29). Research has also examined protective factors such as availability of social support that may moderate the association of stress on adolescents' anxiety and stress symptoms (30). Although no known studies have specifically examined the role of health behaviors such as good sleep, nutrition, and exercise in mitigating stress in military-connected adolescents, studies of military spouses have found that these behaviors may shield against the effects of stress on psychological health (31). Other studies have found that community-level supports buffered negative outcomes, such as for adolescents living in military base housing compared with adolescents not living on base (17, 29).

Importantly, a growing body of work suggests the psychological health of the civilian parent is paramount to their child's adjustment (17, 18, 32, 33). Identifying resilience factors among military-connected youth is critical in developing effective interventions, particularly for youth who have been exposed to higher levels of military stress.

Although adolescent psychosocial functioning has been a fertile subject of research for decades, most studies have been conducted with adolescents outside of military-family contexts. Furthermore, much extant research on military-connected adolescents uses administrative data only [e.g., medical records or Department of Defense (DoD) records] or uses parent reports of youth experiences, health, and well-being as a proxy for adolescent functioning rather than utilizing self-report data from adolescents themselves. This approach may not accurately gauge youths' thoughts, feelings, and behaviors, particularly among older youth who are able to spend more time outside of the home. The conflicting reports between parents and adolescents has contributed to mixed findings regarding the impact of military operational stressors on adolescents' health and well-being, with some research showing that these youth are generally more resilient than their civilian peers (34, 35), while other research has found that military-connected youth exhibit higher rates of depression (36), substance use (37), suicidality (38), difficulties with peers (39), and lower academic performance (17, 18) than their civilian peers.

After nearly two decades of sustained warfare, military and veteran families have made considerable sacrifices in service to the U.S. In response, a report from the Joining Forces Interagency Policy Committee describes the commitment of the Biden-Harris Administration to U.S. military families (40) to address a major knowledge gap by supporting the development of a large-scale, representative study designed to uncover risk and protective factors related to psychosocial outcomes of military-connected youth. This study will enable DoD to understand critical issues facing military-connected youth and their families, provide key insights into modifiable risk and protective factors to inform military programs and services aimed at military-connected youth and their families, identify knowledge gaps pertinent to understudied populations (e.g., adolescents in minority families and single-parent households), and uncover emerging and specialized needs among families to inform tailored programs. Evaluating the long-term well-being of youth in the military community supports the goals of the Joining Forces initiative to enhance military child education and military family health and well-being.

### 1.1 Overview of the millennium cohort study of adolescent resilience

The Millennium Cohort Study of Adolescent Resilience (SOAR) is part of the larger Millennium Cohort Program (MCP), which includes the Millennium Cohort Study (MCS) of service members and veterans and the Millennium Cohort Family Study (Family Study) of military spouses. The MCS was launched in 2001 with the enrollment of its first panel of participants drawn from a representative random sample of service members. This prospective epidemiological study was designed to evaluate the effects of military service on the long-term health and well-being of U.S. service members (41).

With over a quarter of a million participants, the MCS is now the largest and longest running prospective DoD study and is the only study to follow active duty, Reserve, and National Guard personnel from all service branches during and after their military service (42). In addition, the Family Study was established in 2011 as a complementary effort to assess the effects of military experiences on the health and well-being of the spouses and children of service members who enrolled in the MCS that same year (43, 44). Leveraging existing MCP infrastructure supports, SOAR was established in 2022 and designed by a multidisciplinary team of investigators at the Naval Health Research Center (NHRC), with survey development guided by subject matter experts across DoD and academia. SOAR uses a multi-informant design to survey MCS participants, their children aged 11–17 years, and their other parent, if applicable, on topics related to adolescent psychosocial adjustment and physical health, academic achievement, and educational or career aspirations. SOAR survey data will be linked with MCS, and when possible, with Family Study and military administrative and medical health records. Moreover, adolescent participants will be surveyed approximately every 18 months until they reach the age of 25 (emerging adulthood stage).

### 1.2 Purpose and aims of SOAR

The primary objective of the study is to assess the impacts and associations of military experiences on the health and well-being of military-connected adolescents. The data collected will have implications for family readiness and delivery of military family-related services and will inform existing and future training or family-focused programs, education efforts, policy development, and clinical practice to best support military adolescents throughout their parent's military service.

SOAR has three overarching research questions:

1. What are the direct associations between military experiences, such as permanent change of station (PCS) relocations, parental separations, and parental injury, and adolescents' psychosocial adjustment and physical health, academic achievement, and educational and career aspirations?
2. What are the indirect associations between military experiences, such as PCS moves, parental separations, and parental injury, and adolescents' psychosocial adjustment and physical health, academic achievement, and educational and career aspirations through their impact on parents' marital/relationship quality, parent–adolescent relationship quality, and parenting behaviors?
3. What are the most salient, modifiable risk and protective factors that may explain differences in adolescents' psychosocial development and physical health, academic achievement, and educational and career aspirations?

## 2 Methods and analysis

SOAR is a longitudinal, multi-informant cohort study, including self-report data from military-connected adolescents and from their parent(s). Data from SOAR surveys will be linked to various official DoD data files, including military occupational and medical data records.

### 2.1 Study participants

**Figure 1:**
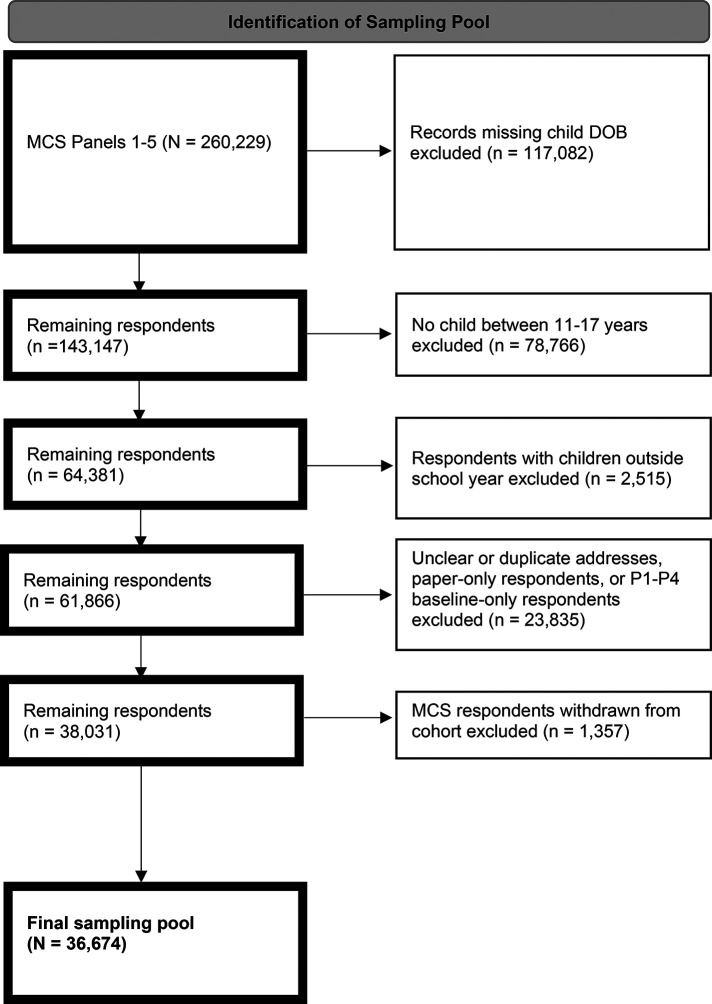
Flow diagram for study of adolescent resilience (SOAR) recruitment. DOB, date of birth; MCS, Millennium Cohort Study; P, panel.

The SOAR sampling frame was drawn from MCS participants enrolled between 2001 and 2021 (see Figure 1).

Participants enrolled in MCS were recruited from a random sample of U.S. military service members on active rosters maintained by DMDC, including individuals from all service branches (i.e., Army, Marine Corps, Navy, Air Force, Space Force, and Coast Guard) and components (i.e., active duty, Reserve, and National Guard). The first panel of participants consisted of service members from a probability-based sample of the entire military population on July 1, 2001. To augment the initial panel, four additional random samples of personnel, with lengths of military service ranging from 1 to 5 years, were enrolled in 2004, 2007, 2011, and 2021, respectively. Over half of the current MCS population are no longer in active duty service. Response rates for enrollment of new panels ranged from 20% to 36%, however, investigations of potential biases in the MCS have found participants to be representative of the U.S. Armed Forces population (45). Service members and veterans enrolled in MCS with an adolescent child between the ages of 11 and 17 years during the 2022/23 academic year according to DMDC records were eligible to participate in SOAR.

Table 1 presents the sociodemographic and military characteristics of service members with adolescent children in the final SOAR study sampling pool (*N *= 38,031). The majority of service member participants invited to SOAR were male (75.2%), aged 25–34 years (46.9%), non-Hispanic White (69.9%), and married (81.2%). Most participants were on active duty (40.9%) and enlisted (i.e., military personnel who serve under officers; 74.4%). Further, most of the sampling frame participants were in the Army (44.7%), followed by the Air Force (30.7%), Navy (15.1%), Marine Corps (7.8%), and Coast Guard (1.9%).

**Table 1:** Characteristics of service member respondents from the study of adolescent resilience (SOAR) sampling pool (*N* = 38,031).

Service member characteristics	*N* (%)
Sex	
Male	28,584 (75.2)
Female	9,447 (24.8)
Age, years	
18–24	27 (0.1)
25–34	17,833 (46.9)
35–44	13,660 (35.9)
45 and older	4,498 (11.8)
Missing	2,013 (5.3)
Race and ethnicity	
American Indian	533 (1.4)
Asian/Pacific Islander	1,541 (4.1)
Black, non-Hispanic	3,841 (10.1)
White, non-Hispanic	26,601 (69.9)
Hispanic	3,048 (8.0)
Other	448 (1.2)
Missing	2,019 (5.3)
Marital status	
Single, never married	2,026 (5.3)
Married	30,898 (81.2)
Separated	1,351 (3.6)
Divorced	3,684 (9.7)
Widowed	72 (0.2)
Service branch	
Army	16,985 (44.7)
Navy	5,727 (15.1)
Marine Corps	2,951 (7.8)
Air Force	11,662 (30.7)
Coast Guard	706 (1.9)
Rank	
Enlisted	28,304 (74.4)
Warrant officer	1,170 (3.1)
Commissioned officer	8,557 (22.5)
Military status	
Veteran	14,445 (38.0)
Active duty	15,572 (40.9)
Reserve or National Guard	8,014 (21.1)

### 2.2 Materials and equipment

MCS participants were invited to the study via email and postal mailings containing a link to the web-based survey. Once the MCS participant logged into the web survey hosted on the study website (www.millenniumcohort.com/soar), they completed consent and HIPAA forms (parent and child), and confirmed having a child aged 11–17 years. If the MCS participant indicated having more than one eligible adolescent child, one child was randomly selected via a pre-programmed algorithm as the focal child. Child questions were then answered only about this focal child. Upon completion of the survey, the MCS parent was asked to provide complete names and email addresses for the focal adolescent and the other primary parent so that they could be invited to join the study. Referred family members were each provided with a unique subject identification number and password associated with the appropriate web survey (i.e., adolescent or other primary parent) via email and postal mailings.

**Figure 2:**
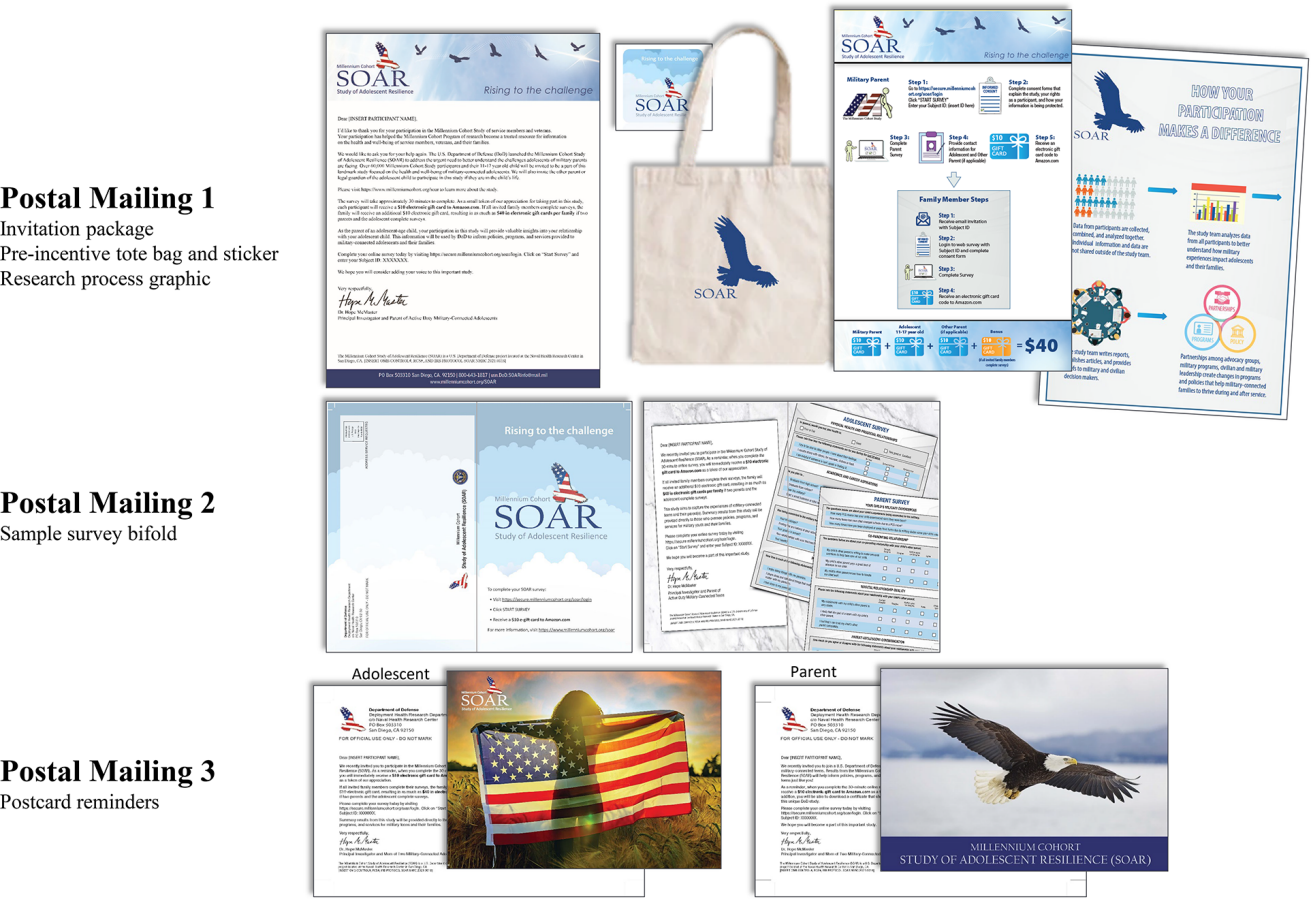
Study of adolescent resilience (SOAR) recruitment materials, including three separate postal mailings.

Given the complexity of a multi-informant design, considerable attention was paid to creating recruitment materials to enhance participation rates (Figure 2). The study name (SOAR), tag line (Rising to the Challenge), and logo (soaring eagle) were designed to evoke positive thoughts of strength and empowerment, in addition to making postal and email communication easily recognizable. The theoretical basis for designing each contact drew heavily from social exchange theory (46), as presented by Dillman et al. (47). MCS participants invited to the study were sent a postal mailing containing a tote bag and sticker with the study logo (pre-incentives), an illustrated explanation of the research highlighting the $10 Amazon gift code post-incentive for each family member, and a personalized letter from the SOAR principal investigator underscoring the importance of participation. The second postal mailing provided a sample of the survey, which highlighted questions from various sections of the adolescent and parent surveys, to legitimize the study and provide survey content without needing to log into the survey site. The third postal mailing included a postcard branded with the study logo, inspirational images, and a note from the principal investigator reiterating the benefits of participation (i.e., helping military families, monetary incentive, adolescent certificate of completion). In addition, study invitation emails were sent to parents and adolescents, and nonresponder and partial responder reminder emails were sent weekly to prompt survey completion. To encourage response from adolescents, referring parents were sent weekly emails requesting that they remind family members to complete their surveys. A “thank you” email was sent to everyone upon completion of the survey, along with a $10 Amazon gift code. Adolescents were emailed an additional $10 Amazon gift code when all referred family members completed their surveys. All correspondence provided contact information for the study team, survey login information, and the survey web address.

Recruitment of study participants was also facilitated by the study website, which augmented information sent in email and postal mailings. The website included details about the confidentiality of survey responses, data security, frequently asked questions, contact information, and survey approval numbers in order to alleviate any possible concerns regarding the legitimacy of the research.

### 2.3 Retention of participants

Between survey collections cycles, participants will be sent emails and postal mailings with study findings, press coverage, and information on how the study is being used to directly inform policies and programs for military and veteran families. These strategies have been successfully used by the MCP over the last 20 years to engage participants and to maintain current postal addresses.

### 2.4 Survey instruments and supplementary databases

**Figure 3:**
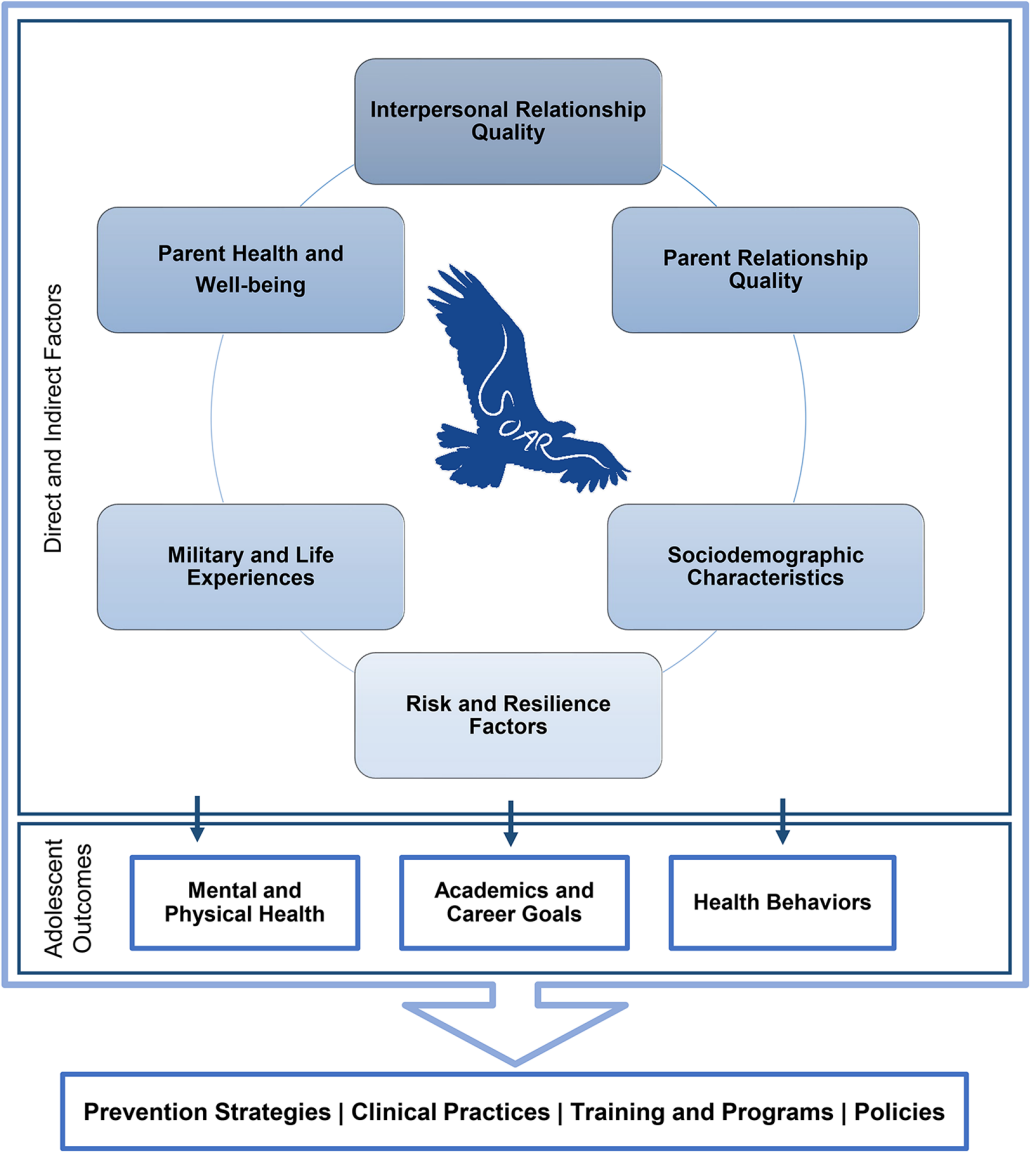
Study of adolescent resilience (SOAR) conceptual model.

The SOAR adolescent and parent questionnaires included approximately 60 and 75 questions, respectively. Some items across both questionnaires included multiple components (i.e., item matrices, follow-up items) and skip logic. Specific questions within the survey were drawn from a conceptual model adapted from the Family Study spouse and child models (48). The SOAR model comprises six content areas theorized to link to military-connected adolescent outcomes in different capacities: (1) interpersonal relationships, (2) parent relationships, (3) parent health and well-being, (4) military and life experiences, (5) sociodemographic characteristics, and (6) risk and resilience factors (see Figure 3).

The surveys included items assessing participants' demographic characteristics and service history for military parents, and various aspects of participants' social support networks, physical and mental health, and military experiences. Adolescent and parent questionnaires were divided into eight specific domains. Each domain was composed of themes that varied depending on whether the survey was designed for an adolescent (Table 2) or for a parent.

**Table 2:** Key domains and a selection of measures included in the study of adolescent resilience (SOAR) adolescent surveys.

Domain	Theme	Standardized measures
Sociodemographic factors	Age, sex, gender identity, sexual identity, ethnicity, race, birthplace, employment	Youth Risk Behavior Survey (YRBS) 2021 (49), Child Trends (50), Joint Advertising Market Research & Studies (51), National Survey on Drug Use and Health (NSDUH) (52)
Physical and psychological health	Overall health, body esteem, psychosocial adjustment, resilience, mental health	NSDUH (52), Body-Esteem Scale for Adolescents and Adults (53), Strengths and Difficulties Questionnaire (54), Patient Health Questionnaire (PHQ-2) (55), Generalized Anxiety Disorder Scale (GAD-2) (56)
Health behaviors	Alcohol use, cigarette use, drug use, sexual behaviors, sleep, physical activity, sedentary behaviors, social media use	YRBS 2021 (49)
Academics and extracurricular activities	School attendance, grade level, grades, school engagement, teacher quality, school environment, military peers at school, school-based mental health services, extracurricular activities, educational/career aspirations	YRBS 2021 (49), NSDUH (52), National Assessment of Educational Progress (57), RAND Deployment Life Study (58)
Military and general life experiences	Permanent change of station moves, military-related school changes, military-related separations from parents, impact of military experiences, support of parent military service, caregiving	Millennium Cohort Family Study (48), DMDC Contingency Tracking System, National Alliance for Caregiving Youth Study (59)
Parent–adolescent relationship	Rules and boundaries, parental supervision, autonomy, decision making, relationship quality, communication, conflict	NSDUH (52), Monitoring the Future (60–62), Maryland Adolescent Development in Context Study (MADICS) (63) Patient-Reported Outcomes Measurement Information System (64)
Youth risk and resilience factors	Gender expression, self-esteem, character, sociopolitical discussions, civic modeling, religiosity	MADICS (63), Rosenberg Self-Esteem Scale (65), Positive Youth Development Student Questionnaire Short Version (66, 67), Youth Civic and Characters Measures Toolkit (68), NSDUH (52)
Prosocial relationships	Bullying victimization and perpetration, peer relationships, romantic relationships, sibling relationships	Olweus Bully/Victim Questionnaire (69), Network of Relationships Inventory–Relationship Quality Version (unpublished measure)

Additional themes were included in the parent questionnaire (Table 3) relative to the adolescent questionnaire to reduce survey burden for adolescents and to increase the accuracy of information associated with objective questions (e.g., services used, household composition). In this way, the SOAR adolescent questionnaire was streamlined to improve usability and to conserve adolescent attentional resources.

**Table 3:** Key domains and a selection of measures included in the study of adolescent resilience (SOAR) parent surveys.

Domain	Theme	Standardized measures
Sociodemographic factors	Military status, military characteristics, number of children and child characteristics, child military life exposure, contact with child, age, sex, gender identity, sexual identity, ethnicity, race, birthplace, education, employment status, income, marital status, household composition	Defense Enrollment Eligibility Reporting System, Millennium Cohort Family Study (MCFS) (48), Joint Advertising Market Research & Studies (51), Child Trends (50)
Physical and psychological health	Health conditions, general health, mental health, treatment, adolescent health	MCFS (48), Military Health System Data Repository, Patient Health Questionnaire (PHQ-2) (55), Generalized Anxiety Disorder Scale (GAD-2) (56), Post-Deployment Health Assessment (PDHA) (70), Strengths and Difficulties Questionnaire (54), Youth Risk Behavior Survey (YRBS) 2021 (49), Survey of Active Duty Spouses 2017 (71)
Parent military and general life experiences	Deployment experiences, military pride, military services, military support, military satisfaction, stressful life events, adverse childhood experiences, caregiving	MCFS (48), PDHA (70)
Health behaviors	Alcohol use, cigarette use, sleep, physical activity, sedentary behaviors	MCFS (48), CAGE (72), YRBS 2021 (49)
Parents’ relationship	Parenting alliance, marital/relationship quality, marital/relationship instability, counseling	Parenting Alliance Inventory (73), Quality of Marriage Index (74), MCFS (48), Dyadic Trust Scale (75)
Adolescent academics and extracurricular activities	School attendance, school type, absences, school contact, special education, extracurricular activities, adolescent utilization of military-sponsored programs	YRBS 2021 (49), Survey of Active Duty Spouses 2017 (71), National Survey of Children's Health (NSCH) (76), MCFS (48)
Adolescent military and general life experiences	Permanent change of station (PCS) moves, school changes and adjustment, separations due to military obligations (deployments, trainings) and adjustment, deployment/reunion/transition-related stress, military discharge and separation, childhood trauma, adolescent impact of military experiences and military resilience, caregiving	MCFS (48), DMDC Contingency Tracking System, PDHA (70), Adverse Childhood Experiences Questionnaire (77), National Alliance For Caregiving Youth Study (59)
Parent–adolescent relationship	Monitoring, supervision, discipline, praise, communication, conflict, stress	Alabama Parenting Questionnaire–Short Form (78), NSCH (76), NSDUH (52), MCFS (48)

Data on adolescent health and well-being were reported by both parents (as applicable) and by the adolescent themselves (i.e., triangulated), and they may be linked with various official DoD data files, including military and medical data records. These data include medical care services (medical diagnostic codes, vaccinations, and pharmaceutical prescriptions) provided to the service member, their spouse, and their adolescent child at military treatment facilities or through the military insurance program (TRICARE). Likewise, data on service members' deployments, occupations, injuries, environmental exposures, and other military events (e.g., disciplinary actions, promotions, and separation) can be investigated with respect to adolescent outcomes. All told, the primary data collections along with administrative data will produce a comprehensive data set to elucidate the effects of military family life on the health and psychosocial well-being of adolescents.

### 2.5 Analytic approach

Planned analyses involve a mix of univariate, multivariable, and multivariate statistics, including descriptive statistics to describe sample characteristics, group comparison analyses, multiple regression models, and structural equation modeling to test research questions. Multilevel (hierarchical) models will be used to estimate relationships between study constructs across levels while accounting for between, within, and bidirectional effects at the family unit level (79). Analyses will center on five primary research aims: (1) determine the unique contribution of military-specific experiences and stressors on adolescent academic and health outcomes; (2) identify the unique contribution of parental military experiences to parenting behaviors, and the impact of parental military experiences on adolescent outcomes through parenting behaviors; (3) explore group-specific differences in adolescent outcomes (i.e., unique influence of parent gender, dual-military families, other family structures); (4) determine the added effects of risk and protective factors above and beyond military exposures on adolescents' outcomes, accounting for between, within, and bidirectional effects at the family level; and (5) explore differences between adolescents who participate in vs. do not participate in military-sponsored programs (e.g., Junior Reserve Officers' Training Corps) activities. Further, data will be compared across baseline and follow-up surveys to examine changes over time in self-reported psychosocial adjustment, physical health, and academic and career outcomes. Administrative datasets will be linked with SOAR data to investigate health outcomes captured by medical records and military experiences captured by personnel data. Reports and briefings will be generated and provided to DoD stakeholders, and infographics will be posted on the SOAR and MCS websites to ensure timely dissemination of study results and to promote study engagement.

### 2.6 Response rates

**Figure 4:**
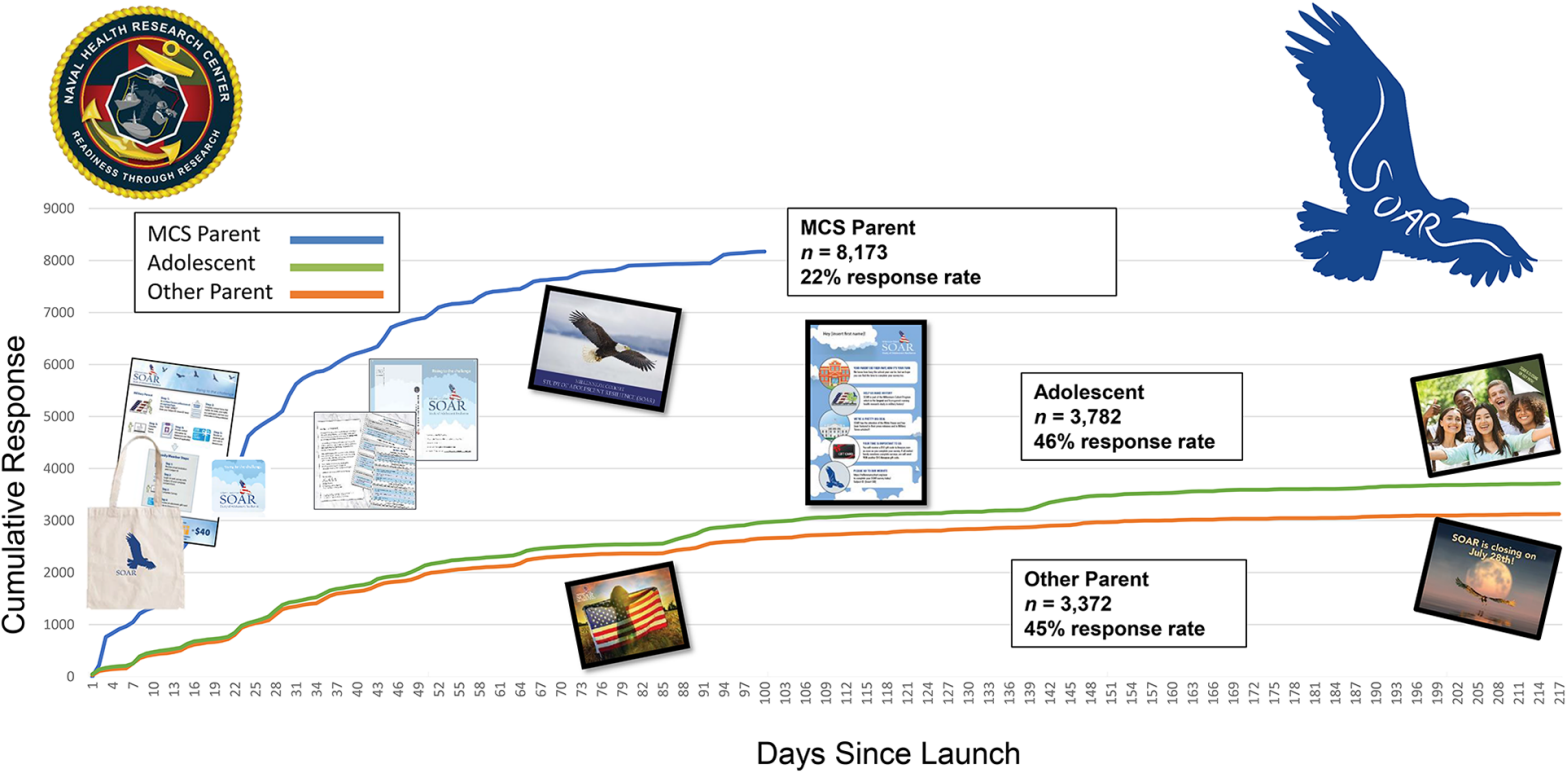
Study of adolescent resilience (SOAR) response rates for millennium cohort study (MCS) parent, adolescent, and other parent.

The survey cycle remained open from November 22, 2022, to July 28, 2023, in order to facilitate answering questions related to academics and extracurricular activities that typically coincide with the school year. Figure 4 provides response rates over the survey cycle and the timing of study postal mailings.

Response rates were typical of large computer-administrated surveys, with small upticks in response around pre-incentive contacts. Once approximately 8,000 military parents were recruited, all recruitment efforts were directed toward completing dyads and triads to include the military adolescent and their other parent, if applicable.

### 2.7 Participant characteristics

Data were collected from 14,826 participants (see Table 4 for participant characteristics). Across all participants, most identified as White and non-Hispanic. Of the 7,870 MCS parents, 69.3% identified as male, and 30.3% identified as female. Most MCS parents were cisgender (i.e., sex assigned at birth is the same as their gender identity; 98.5%), and fewer than 1% identified with a gender different from their sex assigned at birth. The majority of MCS parents identified as heterosexual (94.9%); 0.6% of MCS parents identified as gay or lesbian, 2.1% identified as bisexual, 0.4% identified with a different sexual identity, and 1.0% preferred not to answer. Most MCS parents were married at the time of their participation (80.4%). Most MCS parents were active duty (58.6%), enlisted (62.8%), and served in the U.S. Army (43.1%). In addition, 1.5% of parent participants were in dual active duty or National Guard/Reservist relationships.

**Table 4:** Participant characteristics for millennium cohort study (MCS) parents, other parents, and adolescents.

	MCS parent		Other parent		Adolescent	
	*N*	%	*N*	%	*N*	%
Total	7,870		3,182		3,774	
Sex						
Male	5,431	69.7	788	24.8	1,890	50.1
Female	2,363	30.3	2,393	75.2	1,884	49.9
Gender identity						
Male	5,404	69.3	779	24.5	1,860	49.3
Female	2,347	30.1	2,379	74.8	1,722	45.6
Transgender, male to female	4	<0.1	3	<0.1	5	0.1
Transgender, female to male	3	<0.1	2	<0.1	39	1.0
Something else	15	0.2	5	0.2	73	1.9
Prefer not to answer	19	0.2	13	0.4	74	2.0
Race^a^						
American Indian	309	4.0	78	2.5	135	3.6
Asian	425	5.5	205	6.4	313	8.3
Pacific Islander	129	1.7	49	1.5	78	2.1
Black	800	10.3	242	7.6	399	10.6
White	6,647	85.3	2,735	86.0	3,328	88.2
Latino						
No	6,519	83.7	2,757	86.7	3,120	82.8
Yes	1,274	16.4	422	13.3	650	17.2
Marital status or parent's marital status						
Single, never married	380	4.9	86	2.7	145	3.9
Married	6,220	80.4	2,896	91.0	3,181	84.9
Separated	149	1.9	46	1.5	64	1.7
Divorced	965	12.5	153	4.8	345	9.2
Widowed	20	0.3	0	0.0	10	0.3
MCS parent service branch						
Army	3,386	43.1	1,328	41.8	1,589	42.1
Navy	1,219	15.5	555	17.5	624	16.6
Marine Corps	525	6.7	235	7.4	273	7.2
Air Force	2,558	32.5	998	31.4	1,215	32.2
Coast Guard	174	2.2	64	2.0	70	1.9
MCS parent rank						
Enlisted	4,938	62.8	1,815	57.1	2,200	58.3
Warrant officer	208	2.6	76	2.4	95	2.5
Commissioned officer	2,723	34.6	1,290	40.6	1,478	39.2
MCS parent military status						
Active duty	4,609	58.6	1,897	59.6	2,203	58.4
Reserve or National Guard	3,260	41.4	1,284	40.4	1,570	41.6
MCS separated from service						
No	5,380	68.4	2,147	67.5	2,511	66.5
Yes	2,490	31.6	1,035	32.5	1,263	33.5

Frequencies and percentages may not sum to the total responding population or 100% due to missing values, which were excluded from this table.

Values are preliminary and have not undergone cleaning for discrepancies or outliers.

^a^ Race options are not mutually exclusive because participants were allowed to answer more than one race.

About three quarters of other parents (*n* = 3,182) identified as female (75.2%), and 24.8% identified as male. Most other parents identified as cisgender (99.9%), and less than 1% identified with a gender different from their sex assigned at birth. The majority of other parents identified as heterosexual (94.72%), and a small proportion of other parents identified as gay or lesbian (0.5%), bisexual (2.8%), or a different sexual identity (2.8%), or indicated they preferred not to answer (1.4%). Most other parents were married at the time of study participation (91.04%). Of the 3,774 adolescents, 49.3% identified as male, 45.6% identified as female, and less than 1% identified with a gender different from their sex assigned at birth. Among adolescents, 63.34% identified as heterosexual; 22.5% identified as gay or lesbian, bisexual, a different sexual identity, or were unsure about their sexual identity; and the remaining either skipped the question or indicated that they did not understand the meaning of the question.

### 2.8 Anticipated impact

SOAR is the most comprehensive study of military-connected adolescents undertaken by the DoD. It is expected to provide key insights on modifiable risk and protective factors to inform military programs and services for military-connected youth and their families and to identify knowledge gaps to guide future research. SOAR's multi-informant design, combined with longitudinal follow-up of adolescents into emergent adulthood, is a powerful tool for understanding adolescent health and well-being.

## 3 Discussion

Adolescence is a challenging developmental period for any child, perhaps more so for those in military families. It is critical to identify the unique challenges faced by military-connected adolescents from their perspectives, and to provide them with essential supports and resources to ensure positive psychosocial health and well-being. This study is the first of its kind to address the impact of military family life on military-connected adolescents through a comprehensive data collection effort that includes longitudinal self-report data from adolescents, their parents, and administrative and medical data in concert. Research findings will improve empirical understanding of the impact of military life on adolescents and the nature of associations between military-specific experiences and psychosocial health and well-being. Further, this research will assess prominent modifiable risk and protective factors that may elucidate differences in psychosocial development and physical health, academic achievement, and educational and career aspirations.

Data from this research study will be used to address the association between parents' military exposures and their adolescent children's health and well-being. Important conditions of this association, such as the quality of parent relationships and how these relationships fluctuate over time, will reveal salient risk and protective factors that differentiate positive and negative outcomes among adolescents at different developmental stages. The capability to link SOAR survey data with prior deployment records, longitudinal MCS survey data, and parent and child medical records will provide a longer-term perspective of the impact of military risk factors on adolescent and parent current well-being. For example, medical records could be used to examine the impact of PCS moves on adolescent healthcare service utilization and DoD personnel records could be used to examine the impact of deployment frequency and length on parent-child relationships. Likewise, the inclusion of all branches, veterans, Reserve and National Guard member families, and dual-military couples in this study will allow for examination of these important yet understudied groups.

Notably, a longitudinal research approach is the only mechanism by which we can temporally assess the causal relationships between military-specific experiences and health and well-being outcomes among military-connected adolescents. This is imperative in military contexts, given the numerous transitions and acute but consistent challenges faced by military families. This approach allows for nuanced understanding of adolescent well-being across experiences like PCSs and changing schools, parent deployments and pre- and postdeployment phases, parent changes in rank or other employment factors over time, and parent injuries or separation from the military. Longitudinal data collection over a series of time points will allow for analyses measuring change over time and testing causal hypotheses and behavioral trajectories. This is particularly important during adolescence, given the vast developmental milestones that occur across time and the consequences of these changes for future adult outcomes. Ultimately, this line of research will provide a foundation for evidence-based interventions to support military-connected adolescents, thereby strengthening military families and promoting well-being and readiness among U.S. warfighters.

## 4 Ethics and dissemination

To ensure the welfare of study participants, regulatory approval was received from the NHRC Institutional Review Board (NHRC.2021.0018), per DoD requirements. Survey approval was received from the Office of Management and Budget (#0704-0635). Data access and data management procedures are compliant with DoD requirements for the protection and confidentiality of research participants. Findings from this study will be disseminated through national and international peer-reviewed journal publications, the Defense Technical Information Center, and academic and military conferences. Findings from this research study will be leveraged by key military family stakeholders to inform policies and programs geared toward supporting military-connected youth.

## Author disclaimer

HM is an employee of the U.S. Government. This work was prepared as part of her official duties. Title 17, U.S.C. §105 provides that copyright protection under this title is not available for any work of the U.S. Government. Title 17, USC, §101 defines a U.S. Government work as work prepared by a military service member or employee of the U.S. Government as part of that person's official duties. Report No. 23-62 was supported by the U.S. Navy Bureau of Medicine and Surgery under work unit no. 60002. The views expressed in this article are those of the authors and do not necessarily reflect the official policy or position of the Department of the Navy, Department of Defense, nor the U.S. Government. The study protocol was approved by the Naval Health Research Center Institutional Review Board in compliance with all applicable federal regulations governing the protection of human subjects. Research data were derived from approved Naval Health Research Center Institutional Review Board protocol NHRC.2021.0018.
